# Pattern of lateral neck metastases in N0 papillary thyroid carcinoma

**DOI:** 10.1186/1471-2407-11-8

**Published:** 2011-01-11

**Authors:** Vincent Patron, Cécile Bedfert, Guy Le Clech, Karine Aubry, Franck Jegoux

**Affiliations:** 1 Department of Head and Neck Surgery, Rennes University Hospital Center, 2 rue Henri Le Guillou; 35000 Rennes, France; 2 Department of Head and Neck Surgery, Limoges University Hospital Center, 2 avenue Martin Luther-King, 87000 Limoges, France

## Abstract

**Background:**

Indication and extent of lateral prophylactic neck dissection (PLND) in papillary thyroid carcinoma (PTC) is very controversial.

**Methods:**

We retrospectively analysed 131 patients who underwent thyroidectomy and prophylactic lateral neck dissection from level II to V for PTC.

**Results:**

140 PLND were performed. The occult lymph node metastases (OLNM) overall rate was 18.6%. The incidence of node involvement was 10% at level III and 6.4% at level IIa. Level IV and level Vb were both concerned by 5.7% OLNM. Only 2.9% of level IIb contained OLNM. None of the level Va ND revealed OLNM.

**Conclusions:**

OLNM from PTC occurs commonly in level IIa, III, IV and Vb. Incidence in other levels is low. For surgeons that usually perform PLND, we believe that a selective neck dissection of levels IIa, III, IV and Vb in N0 neck PTC is sufficient for the clearance of occult metastases.

## Background

The management of papillary thyroid carcinoma (PTC) with no clinical nodes remains controversial. Occult lymph node metastases (OLNM) occur in 21 to 90% of patients [1-3], but their role as a prognostic factor for recurrence or death has not been clearly established. There has been recent consensus that in N0 microcarcinomas the best management may be that of a wait-and-see policy. However, the place for central and lateral neck dissection for tumours of more than 1cm in diameter, without clinical lymph node involvement remains controversial.

Extensive radical neck dissection and modified radical neck dissection (ND) leads to an increase in morbidity, mainly due to spinal accessory nerve (SAN) injury which may result in shoulder pain and dysfunction. Moreover, the use of radioactive iodine has allowed successful non-operative management of low-volume cervical disease [4,5]. Thus, many surgeons do not systematically perform prophylactic lateral neck dissection (PLND), while others routinely proceed to modified radical neck dissection (MRND) [6].

Decisions regarding the extent of lymphadenectomy required for N0 PTC should be based on predictable drainage patterns. A better understanding of node distribution could be critical to achieving more selective neck dissection. This field has been little investigated in PTC compared to other head and neck carcinomas and a recent approach involving sentinel biopsy has not yet answered to this question [7].

To study the incidence, extent and spread of lateral node metastasis in PTC we retrospectively reviewed data from patients in our institution treated with systematic prophylactic neck dissection.

## Methods

Medical records of 631 patients who underwent a surgery for thyroid carcinoma in our institution from January 1974 to December 2006 were reviewed. Patients included had criteria of a histological diagnosis of papillary thyroid carcinoma in previously untreated tumours of greater than 10mm with no evidence of node involvement on clinical examination and US imaging. Dissection of at least levels II through V was required for inclusion.

A review of the clinical records and pathological reports was conducted to ascertain the prevalence and distribution of cervical metastases according to neck level. Data from 16 ND were excluded because of missing histological or surgical reporting, leaving 140 PLND in 131 patients to be analyzed.

Total thyroidectomy was the standard procedure. Ipsilateral MRND including levels II, III, IV and V with or without level I was performed if the lesion was located in only one lobe. A bilateral MRND was performed if the lesion was located in the isthmus or disease was present in both lobes. All fascial and nodal tissues contained in level II to V were routinely removed. Those of level Ib were dissected depending upon the surgeon's choice at the time. The spinal accessory nerve (SAN), the internal jugular vein and the sternocleidomastoid muscle were systematically preserved. All patients underwent a systematic level VI (central compartment) per-operative exploration. In cases with suspicious central node involvement, a full dissection to level VI was performed: all fat and fascia between the trachea and the common carotid artery were removed after identification of the inferior laryngeal nerve (paratracheal ND). Additional pretracheal and pericricoid ND were also performed. All neck levels were marked and sent for pathological analysis with conventional haematoxylin-eosin staining. No immunochemistry was performed.

Lymph nodes levels were retrospectively defined using the agreed nomenclature of the AAO-HNS [8].

## Results

Of the 131 selected patients, 102 were women (78%) and 29 were men (22%) with a mean age of 38.3 years [range 12-67 years]. Total thyroidectomy was performed in 126 patients (96%) and lobectomy in five patients (4%). Tumour size is resumed in table 1.

**Table 1:** Patient demographics

Characteristics	No. Patients (%)
Number of patients	131
Sex	
Male	29 (22)
Female	102 (78)
Average age (range)	38.3 y (12-67 y)
Tumor size	
10mm ≤ T ≤ 20mm	87 (66)
20mm < T ≤ 40mm	30 (23)
>40mm	8 (6)
Unknown	6 (5)
Surgical procedure	
Total thyroidectomy	126 (96)
Lobectomy	5 (4)
Lymph node dissection	
Unilateral	122 (93)
Bilateral	9 (7)

Of the 140 PLND performed in these 131 patients, nine patients required bilateral dissection. Lateral neck dissection was associated with a level VI full dissection in 37 cases (26%). 98 of the 140 PLND (70%) included a level Ib dissection. A mean of 13.5 lymph nodes per PLND [range 3-44 lymph nodes] was analyzed (1574 lymph nodes in 117 PLND).

The rate of positive findings in lateral neck dissection was 18.6% (26 of the 140 PLND). The average number of invaded nodes was 2.6 [range 1-8 OLNM] per positive neck (68 OLNM in 26 PLND).

**Figure 1:**
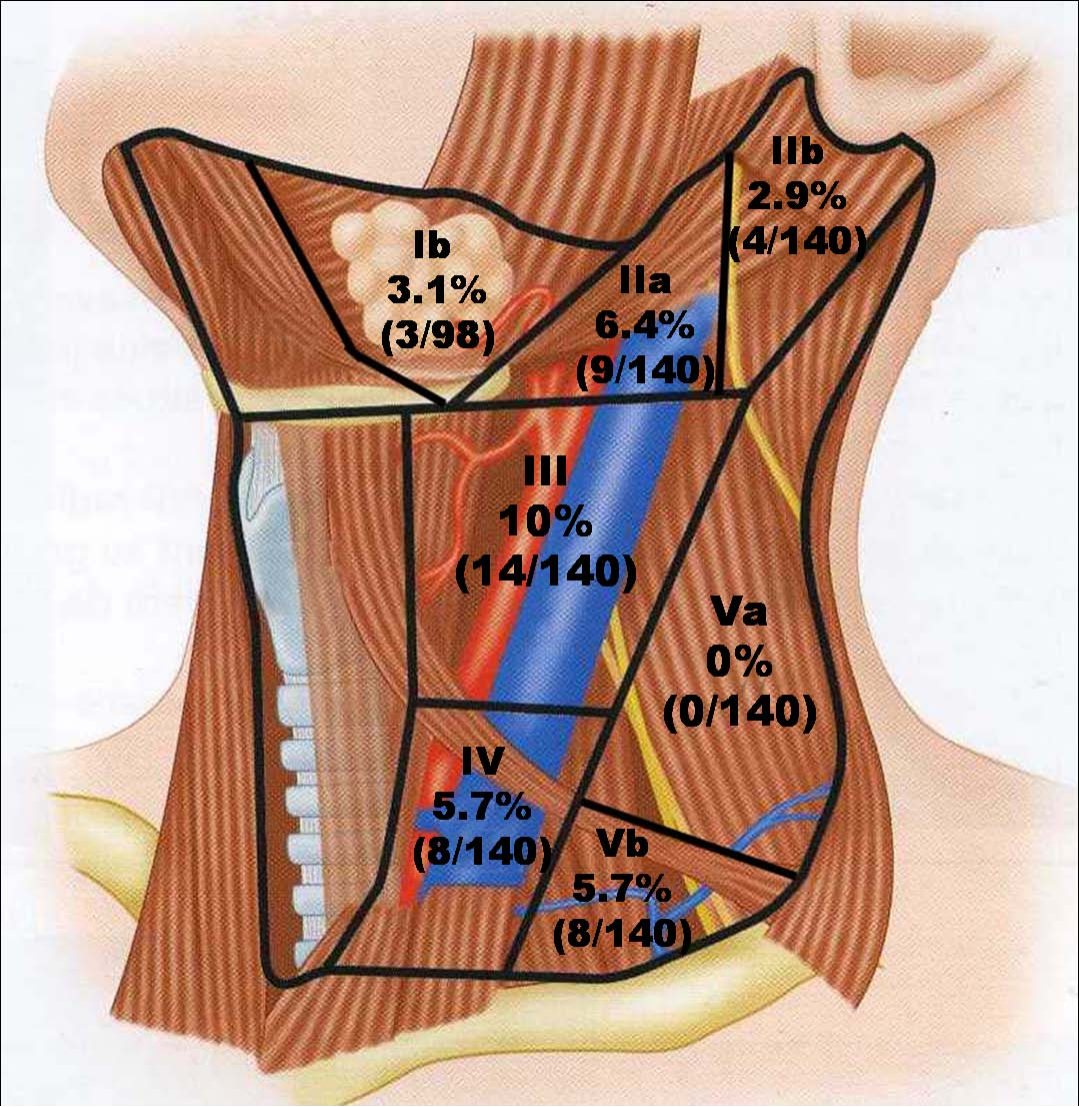
**Percentage of metastases by level (numbers in parentheses indicate number of ND specimens with OLNM/total number of ND specimens)**.

The distribution of OLNM is reported in table 2. The highest incidence of OLNM in the lateral compartment was found in level III dissections, with 14 neck specimens (10%) demonstrating OLNM (Figure 1). Nine specimens revealed OLNM at level IIa (6.4%), eight at level IV (5.7%) and eight at level Vb (5.7%). Of the 98 levels Ib dissections, three contained OLNM (3.1%). Only four (2.9%) level IIb dissections contained OLNM. None of the level Va specimens revealed any OLNM.

**Table 2:** Distribution of OLNM in 26 positive ND

Level	N0
III	4
Vb	4
IIa + III	3
Ib	2
IIa	2
IV	2
IIb + III	2
IIa + III + IV	2
IV + Vb	1
Ib + III + IV	1
IIa + III + Vb	1
IIb + IV + Vb	1
IIa + IIb + III + IV + Vb	1
Total	26

In 14 neck specimens (53%) OLNM only occurred at one level: Ib (n = 2), IIa (n = 2), III (n = 4), IV (n = 2) and Vb (n = 4), whereas no level IIb had any isolated metastases. In 12 neck dissections (47%), OLNM were found at multiple levels. Level IIb metastases were associated with metastases with level IIa in 1 case, level III in 2 cases and with level IV and Vb disease in 1 case. Level IIa metastases, in multilevel involvement, were always associated with level III metastases (7 of 7 specimens). Most of the multilevel involvements including level Vb OLNM (3 of 4 specimens) had level IV OLNM. Level Ib was associated with level III and IV involvement in only one case.

Analysis of patients who had undergone a fully comprehensive neck dissection of lateral and central compartments, revealed OLNM in both central and lateral compartments in five ND (13.5%), in central compartment alone in four ND (10.8%) and in lateral compartment alone in 3 (8.1%).

## Discussion

There is ongoing debate about the appropriate management of neck metastases in PTC. It is agreed that a 'wait-and-see policy' is adequate in N0 microcarcinomas and that therapeutic ND is always indicated in clinically positive lymph node metastasis. The current practice is for systematic central lymphadenectomy as reports suggest that this could reduce lymph node recurrence rates [9], but recent recommendations stated that prophylactic central node dissection may be not necessary for non invasive T1, T2 PTC [10]. Also, the management of N0 lateral disease remains controversial.

There is debate regarding which patients would benefit from neck dissection as well as the extent of the neck dissection [11]. Predictive factors may help better select patients who may be suitable for a 'wait-and-see policy' rather than neck dissection [11], but there is little data concerning those with N0 disease [12]. Moreover, data regarding the prognostic significance of OLNM are contradictory [12,13]. When the decision of ND has been made the current options are for sentinel biopsy, selective or supraselective neck dissection, ipsilateral MRND or bilateral MRND. Either diagnostic or therapeutic, the role of neck dissection is highly dependent on the disease pattern and spread. To our knowledge this study is the first to provide information on the pattern of lateral invasion in N0 PTC using the new nomenclature [8].

Lateral OLNM were present in 18.6% of cases. There is little data on lateral OLNM incidence. Ito found a higher rate of 64% in 1321 patients with PTC [12] whereas Wada reported an OLNM rate of 39.5% in the lateral compartment of patients with microPTC [14]. Microcarcinoma analysis was excluded from our analysis because the question of the extent of neck dissection is of less interest in these cases. OLNM were confined to only one level in 53% of cases, which is within the range of various reports of 32% from Kupferman et al., 39% from Pingpank et al. and 64% from Ducci et al.[15-17] in therapeutic neck dissections. Lee et al. and Sivanandan et al. reported a lower rate of 18% [18,19]. This wide range between studies may be explained by the geographical variability, genetic changes in the tumour tissue (*ras*-, *gsp*-,*p53*- and *p21*-mutations), accuracy in the clinical and imaging diagnosis of the N0 necks and variability of the extent of neck dissection and pathological processes [20].

Levels III, IIa and IV were the most common levels showing OLNM. This is consistent with findings in other previously reported studies of therapeutic ND [15,19] and with the conventional anatomy of lymphatic drainage in the thyroid area. As found in this study, level IIa and III are more likely to be invaded simultaneously [15,19,21] as a consequence of jugular lymphatic vessels crossing these levels with direct drainage of the upper thyroid.

In our study, the neck dissections were extended to the level Ib in 70%, owing to the wide inclusion period. Level Ib was excluded from lateral prophylactic neck dissection along the period of inclusion as in many other reports [13,15]. Level Ib involvement is only 3.1% in this study, which is similar to the rate reported by Roh et al [21]. The increasing reliability of US imaging associated with the low OLNM risk in this level has led us to avoid it in any ND for N0 neck PTC.

In our study, OLNM were found in level Vb (5.7%) but were absent of level Va. Contrary to our results, Roh et al. in a prospective study of 52 patients with therapeutic ND found a higher percentage of LNM in level Va than in Vb (13% vs. 3.7%) [21]. Unfortunately no other study separates results from level Va and Vb. For Kupferman et al. "anecdotal evidence suggests that nodes inferior to the SAN (level Vb) along the transverse cervical vessels harbour thyroid carcinoma metastases whereas the nodes superior to the SAN are less likely to be involved" [22]. The pattern of invasion from inferior parts of the thyroid area toward the level Vb is consistent with the conventional anatomy of lymphatic vessels in their transverse cervical drainage. Level Vb was mostly associated to a level IV involvement which has been reported by Kupferman et al. as the most significant predictive factor of level V OLNM in PTC [22]. The isolated invasion of level Va and IIa are less understood because these levels are not directly involved with lymphatic drainage in physiological conditions. Level IIb OLNM were observed in a few (2.9%) and only in cases of invasion of the surrounding levels IIa and level III. In therapeutic ND, Lee et al. found a low incidence of level IIb LNM (6.8%) although a higher level of III LNM up to 80% [18]. He concluded that level IIb ND should be performed only if there is clinical or radiological evidence of metastases at this level. In prophylactic neck dissections where level III is less involved, level IIb dissection would lead to less likelihood of occult metastases. In our study, no LNM occurred at level Va. These latter observations were associated with a 65% reported rate of shoulder pain and dysfunction after dissection of the SAN [23,24] and are consistent with a strategy of sparing these levels.

Of the population that underwent lateral and central neck dissection, 8.1% had OLNM in the lateral compartment without any involvement of the central compartment. As found in our study, several lateral levels may be involved simultaneously while the central compartment is spared [21]. Sivanandan et al. observed a similar rate of 9.6% and lower rate for microcarcinomas (5.5%) [14,19]. These rates are too high to confirm Machens theory that this is an epiphenomenon in PTC ^23^. Direct lymphatic drainage of part of the thyroid gland toward levels II, III and IV partly explains this phenomenon, however, it is likely that this rate is highly dependent on the extent of the central compartment dissection and also on the quality of histopathological procedure. Serial node sections and immunohistochemistry would decrease the rate of isolated lateral OLNM. In our strategy, the lateral compartment management is never based on the central compartment status.

This retrospective study has several limitations. First, this article does not address the issue of whether or not to perform a prophylactic lateral neck dissection because no comparative study of the impact of neck dissection on recurrence, death and complications of surgery has been performed. The aim of this study was to describe the pattern and spread of occult lateral neck metastases in PTC. Nevertheless, a major data of the present study, the percentage of OLNM (18.6%), is arguing against PLND. It is below the classical threshold of 20% of occult metastases recommended by Weiss et al. to perform elective neck dissection in N0 head and neck squamous cell carcinoma who are known to have more aggressive neck metastases than PTC [25]. Second, this study covers a long period of time (1974-2006), where improvements in imaging may have resulted in differences in the likelihood of detecting preoperative LNM. However, the vast majority of patients (92%) were diagnosed after 1985, at a time where ultrasound was used routinely in our patients.

## Conclusions

The present study provides more information on the pattern of neck lymph node invasion in PTC in N0 disease. The findings suggest that OLNM occurs mainly in level III, IIa, IV, and Vb. The incidence of OLNM after prophylactic ND is much lower than after therapeutic dissection. Invasion of levels Ib, IIb, and Va is a rare event, but this increases when there are OLNM in other surrounding areas. Although this study does not address the issue of whether or not to perform a prophylactic lateral neck dissection, it is of interest to surgeons who usually perform PLND. In these cases we believe that selective neck dissection of levels IIa, III, IV and Vb in cases of PTC with N0 is sufficient for the clearance of occult metastases.

## List of abbreviations

LNM: Lymph node metastases; OLNM: occult lymph node metastases; PTC: papillary thyroid carcinoma; ND: neck dissection; MRND: modified radical neck dissection; PLND: prophylactic lateral neck dissection; SAN: spinal accessory nerve; US: ultrasound; AAO-HNS: American Association of Head and Neck Surgery

## Competing interests

The authors declare that they have no competing interests.

## Authors' contributions

VP: conception and design, acquisition of data, analysis and interpretation of data, final approval.

CB: acquisition and analysis of data, draft of the manuscript, final approval.

GLC: analysis and interpretation of data, draft of the manuscript and revision, final approval.

KA: analysis and interpretation of data, draft of the manuscript, final approval.

FJ: conception and design, analysis and interpretation of data, draft of the manuscript and revision, final approval.

All authors read and approved the final manuscript.

## Pre-publication history

The pre-publication history for this paper can be accessed here:

http://www.biomedcentral.com/1471-2407/11/8/prepub
